# Editorial: Design and construction of microbial cell factories for the production of fuels and chemicals

**DOI:** 10.3389/fbioe.2023.1198317

**Published:** 2023-04-19

**Authors:** Tian-Qiong Shi, Farshad Darvishi, Mingfeng Cao, Boyang Ji, Xiao-Jun Ji

**Affiliations:** ^1^ School of Food Science and Pharmaceutical Engineering, Nanjing Normal University, Nanjing, China; ^2^ Department of Microbiology, Faculty of Biological Sciences, Alzahra University, Tehran, Iran; ^3^ Research Center for Applied Microbiology and Microbial Biotechnology (CAMB), Alzahra University, Tehran, Iran; ^4^ College of Chemistry and Chemical Engineering, Xiamen University, Fujian, China; ^5^ BioInnovation Institute Copenhagen, Denmark; ^6^ State Key Laboratory of Materials-Oriented Chemical Engineering, College of Biotechnology and Pharmaceutical Engineering, Nanjing Tech University, Nanjing, China

**Keywords:** microbial cell factories, fuels and chemicals, synthetic biology, design and construction, metabolic engineering

Green biomanufacturing refers to the advanced production of biofuels, bio-based chemicals and products using industrial biotechnology (Chi et al., 2019; Straathof et al., 2019; Lv et al., 2020; Ye et al., 2023). Microbial cell factories are the central aspect of green biomanufacturing. By introducing heterologous biosynthetic pathways and then modulating complex metabolic flux intracellularly, various fuels and chemicals can be produced in a sustainable manner from renewable carbon resource and in case of high added-value compounds such as artemisinine, they can be produced in an economically feasible manner (Gong et al., 2017; Li et al., 2018; Banner et al., 2021). Compared with chemical synthesis and plant extraction methods, microbial cell factories are not restricted by external factors such as geography, climate and expensive catalysts (Liu et al., 2021; Cho et al., 2022). At the same time, using microbial cell factories for chemical production has been paid more attention due to the inherent advantages including shorter production cycles and higher product yield compared to the previous two methods. In this Research Topic, the design and construction of microbial cell factories for the production of fuels and chemicals were extensively discussed. Ma et al. developed a hybrid system that hired both synthetic biology-based methods (Sbio) and chemical synthesis-based methods (Csyn) to synthesize clinically significant and structurally complex chanoclavine to overcome the limitations of Sbio and Csyn which could boost the production of complex natural products. The model organisms like *Saccharomyces cerevisiae*, *E. coli* and *C. glutamicum* have good potential as microbial cell factories due to their clear genetic background and powerful genetic manipulation tools (Yilmaz and Walhout, 2017). Wang et al. constructed a heterologous biosynthetic pathway in *S. cerevisiae* for mogrol production. The mogrol pathway was divided into three modules and the metabolic flux of these three modules was optimized. Finally, the mogrol titer was increased to 9.1 μg/L which was 455-fold higher than that of the original strain. Guo et al. used *Escherichia coli* as a host cell to produce D-allulose from D-fructose via *in vivo* phosphorylation-dephosphorylation. After replacing the fructose phosphotransferase systems, blocking the carbon flux and introducing an ATP regeneration system, this engineered cell factory cultured in M9 medium with glycerol as a carbon source achieved a D-allulose titer of ≈1.59 g/L and a yield of ≈0.72 g/g on D-fructose. *C. glutamicum* is considered a promising strain for *O*-Acetylhomoserine (OAH) production. Li et al. constructed an efficient clustered regularly interspaced short palindromic repeats/dead CRISPR-associated protein 9 (CRISPR-dCas9) system to identify the key genes in central metabolism and branch pathways associated with OAH biosynthesis. 25.9 g/L OAH was obtained by the engineered strain which provides a better research basis for the industrial production of OAH in *C. glutamicum.* Apart from model organisms, several non-model organisms have also been used as cell factories for the production of high-value chemicals due to their unique advantages (Nielsen, 2019). The industrial production of iturin and fengycin relies on bacterial fermentation rather than chemical synthesis but still suffer from low yields. Wang et al. developed a systematic engineering approach to improve the antifungal activity and biosynthesis of iturin and fengycin in *Bacillus amyloliquefaciens* including increasing precursor supply of the branched-chain amino acids, disrupting sporulation to extend the stage for producing antifungal lipopeptides, blocking siderophore synthesis to enhance the availability of amino acids and fatty acids, and increasing phosphorylated Spo0A by knocking out rap protein. After combined regulation and fermentation optimization, the titer of iturin and fengycin in the final engineered strain reached 31.1 mg/L and 175.3 mg/L in a flask, and 123.5 mg/L and 1,200.8 mg/L in a bioreactor. Li et al. evaluated *Gluconobacter oxydans* ATCC9937 for the production of 2,5-diketo-D-gluconic acid (2,5-DKG) and identified the non-enzymatic browning of 2,5-DKG. After optimizing the fermentation process, the titer of 2,5-DKG increased to 50.9 g/L which provided a basis for further increases in titer of 2,5-DKG. *Yarrowia lipolytica* is an excellent chassis cell due to its ability to utilize a wide range of substrates, accumulate lipids, and resist poor industrial fermentation environment (Larroude et al., 2018; Patra et al., 2021). Zhu et al. and Liang et al. achieved efficient production of lipids and erythritol using *Yarrowia lipolytica* as the host, respectively. Their researches have contributed to a deeper understanding of the *Y. lipolytica* cell factory, laying the foundation for the production of other high-value chemicals. In summary, the selection of suitable host cells is a prerequisite for the efficient production of high-value compounds.

The development of effective strategies is also essential to obtain a robust cell factory. A variety of strategies have driven the rapid development of synthetic biology which enabled us to efficiently edit the genome of host cells to achieve precise regulation and optimization of their genetic components, synthetic pathways, and metabolic networks, and ultimately to build cell factories for the efficient production of high-value compounds (Liu et al., 2022). Ma et al. developed a transposon-mediated random deletion method that allows the random and continuous reduction of *E. coli* genome. Using this strategy, a polyhydroxybutyrate overproduction strain has been obtained with better growth, glucose utilization, protein expression, and a significant increase in electroporation efficiency. Gambacorta et al. used a combinatorial library design and a growth-coupled screening to identify an isobutanol pathway cassette capable of supporting a higher carbon flux to select for both high isobutanol and high ethanol producing strain. Conversion of cheap feedstock to value-added chemicals via green biological processes may provide an attractive approach toward carbon neutrality (Zhu et al., 2020). Methanol is favored for its abundance, low price and highly reducible properties (Wang et al., 2020). Sun et al. reprogrammed *E. coli* metabolism to improve methanol assimilation by combining rational design and adaptive laboratory evolution. Protein engineering is a promising approach to improving enzyme performance, including catalytic activity, stability, selectivity and tolerance (Yang et al., 2019; Yuan et al., 2022). Recently, Huang et al. employed a computer-aided protein design strategy to improve the catalytic efficiency and substrate specificity of the α-L-rhamnosidase from *Thermotoga petrophila* DSM 13995. The best mutant showed a catalytic efficiency increased 209-fold. With the increasing research on cell factories and the growing amount of experimental data, genome-scale metabolic models (GSMs) have been developed for network property analysis, cell phenotype prediction, metabolic engineering guidance, model-driven discovery, evolutionary process exploration, and interspecies interaction identification (Ye et al., 2022). Zhang et al. reconstructed the GEM of *Streptomyces radiopugnans* iZDZ767. The culture conditions were optimized and the potential geosmin overproduction targets were identified, the titer of geosmin reached to 581.6 ng/L. The model iZDZ767 is a powerful tool for analyzing and predicting the metabolic pathways and yields of various products in *S. radiopugnans*, which provides convenient conditions for researchers to study *S. radiopugnans* in the field of systems biology. In recent years, droplet-based microfluidics as an emerging high-throughput screening technology can generate uniformly sized, mutually independent droplets with remarkable features such as high speed, high throughput, and low cost for the screening of a variety of microorganisms, expanding the existing strain library and improving the product titer, which will pave the way for the construction of microbial cell factories (Leavell et al., 2020; Yuan et al., 2022).Zhang et al. reviewed the recent applications of droplet-based microfluidics which provides a useful quick-glance reference for anyone interested in high-throughput screening technology.

The construction of efficient microbial cell factories for the production of high-value compounds is an inevitable trend for sustainable industrial production. This Research Topic not only covers a rich diversity of microbial hosts and high-value chemicals but also introduces several promising metabolic engineering strategies. In general, numerous studies have been conducted to modify host cells to enhance product titers, including the expansion of strain libraries, optimization and regulation of biosynthetic pathways, improvement of enzyme properties, and refinement of gene editing tools. We firmly believe that the rapid development of biotechnology will pave the way for the construction of microbial cell factories in the green biomanufacturing industry.
